# Reviewing as a career milestone: a discussion on the importance of including trainees in the peer review process

**DOI:** 10.1038/s42003-021-02645-6

**Published:** 2021-09-24

## Abstract

In celebration of Peer Review Week 2021, we asked pairs of faculty mentors and their trainees to reflect on their experiences as co-reviewers for manuscripts at *Communications Biology*, and the importance of providing peer review opportunities and recognition to early-career researchers.

**Dr. John Dennehy** is a Professor of Biology at Queens College, City University of New York. He received his Ph.D. in biology from Clark University and was a post-doctoral fellow at Yale University and the State University of New York at Albany before coming to Queens College in 2007.

**Figure:**
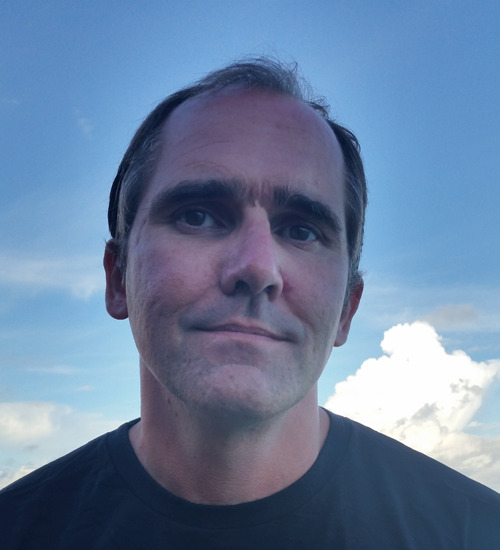
John Dennehy

**Irene Hoxie** is a Ph.D. student in the Dennehy lab at Queens College, City University of New York, where she investigates rotavirus ecology and evolution.

**Figure:**
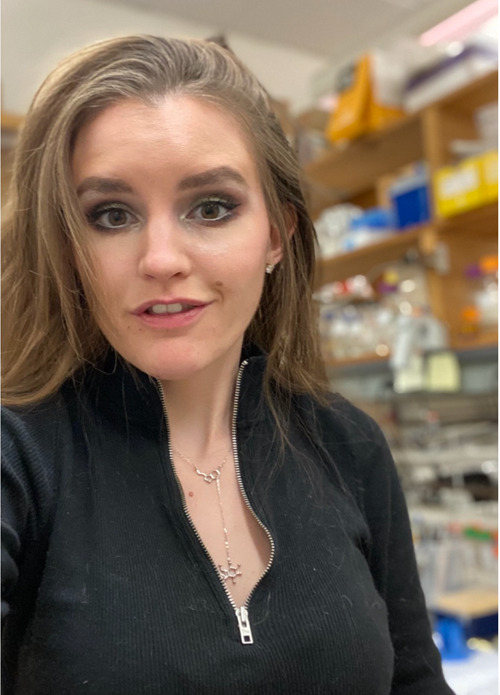
Irene Hoxie

**Dr. Elia di Schiavi** received his Ph.D. in genetics from the University of Naples Federico II, and is currently the head of the *C. elegans* neurobiology lab at the Institute of Biosciences and BioResources in Naples, Italy.

**Figure:**
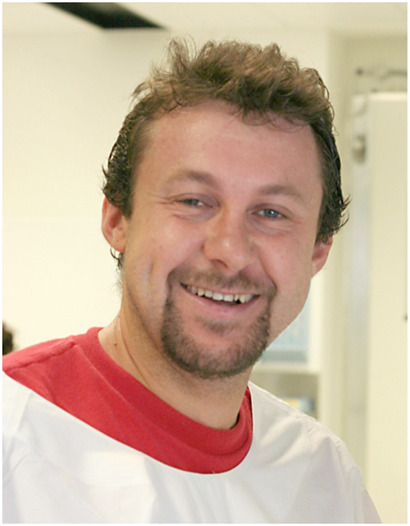
Elia di Schiavi

**Giada Onorato** is a Ph.D. student at the Institute of Biosciences and BioResources, where she is currently working with the di Schiavi lab to investigate genetic background-dependent effects of space-related radiation on the *C. elegans* nervous system.

**Figure:**
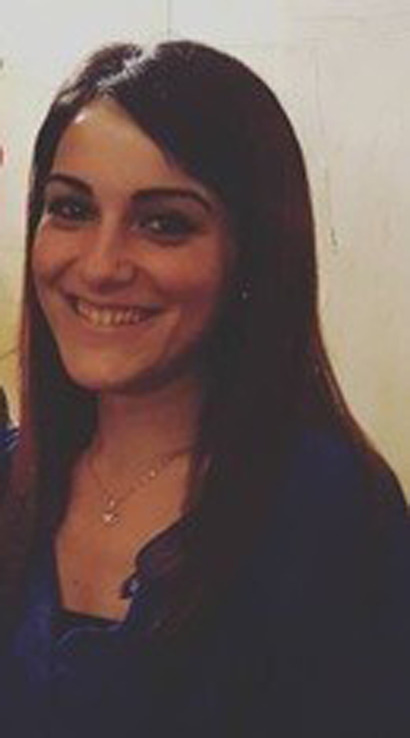
Giada Onorato

John and Irene, please tell us a bit about your research.

**John Dennehy (JD):** The focus of the lab is virus ecology and evolution. The lab works with everything from phages to mammalian viruses. Irene introduced rotaviruses to the lab and made them the focus of her dissertation. She bioinformatically analyzed patterns of genetic exchange and compared the 11 rotavirus segments’ evolution over the past 50 years. In addition, she conducted wet lab experiments on rotavirus reassortment, extracellular vesicle formation, and miRNAs that are carried by rotavirus extracellular vesicles.

**Irene Hoxie (IH):** I’m mostly interested in viral RNA and RNA virus genome evolution, but both John and I started in ecology, evolution, and behavior (EEB) before getting into virology, so I think we both get excited by viral and microbial diversity in general, and anytime those EEB fields overlap with molecular virology.

Elia and Giada, please tell us about your research interests.

**Elia di Schiavi (EDS):** In my laboratory we use the incredible power of the small animal model *C. elegans* to study how neurons are able to cope with different insults of genetic and environmental origin. This fundamental question about how neurons survive for a very long time (decades in some species) is addressed using molecular biology approaches to understand the genetic pathways involved in neuronal degeneration and protection. Moreover, using a high-throughput pharmacological approach, we are also identifying small natural and artificial molecules able to prevent or delay neurodegeneration.

**Giada Onorato (GO):** I am interested in using *C. elegans* as a model organism to understand how environmental conditions and genetic variations can modulate neuron survival and degeneration, including in pathological conditions.

Why do you think peer review is important? Has being a peer reviewer changed your perception of the scientific process?

**EDS:** Albeit imperfect, peer review is the best and only way science can advance and stay protected from non-scientific approaches, thus helping, even recently during the COVID pandemic, science to stay consistent and reliable. Of course, being a reviewer completely affects your perception of the whole scientific process. Only when you know what it means to do something, you truly understand, respect and possibly appreciate it. In my case, I got the feeling that reviewers are human beings, with their values and defects, knowledge and misconceptions, and precious time to dedicate to the review process. This simple message completely changed the way I approach writing papers and the rebuttal process to reviewer comments.

**IH:** I’ve received totally opposite reviews for the same paper (i.e., long lists of difficult-to-address comments, vs. just a couple lines pointing out punctuation errors), so it’s helpful to be exposed to many reviews so you get a better sense of what’s helpful, what’s good science, etc. It also provides a different perspective to be a (small) part of someone else’s science and thought process, without having any kind of bias to the project or people. Usually, we’re just so deep into our own work, so we can’t necessarily see how others might view it. Peer reviewing gives us an opportunity to give an outside perspective.

Why is it important to include trainees in the peer review process and provide them with direct recognition for their involvement?

**EDS:** Including trainees in the review process is pivotal for them (and the whole peer review process), for their education on how to write articles and review them, but also for their professional development. It is very important to stress the importance of giving credit to their work, which is one of the most important rewards that scientists achieve in their career (at least in my opinion). So, it’s important to give a clear message to early-career researchers that reviewing is an important aspect of science, that it is a difficult and long task, but their job will be somehow acknowledged. Of course, there’s also the possibility of adding this experience to build a more appealing CV.

**GO:** Allowing young researchers to participate in the process of reviewing scientific work can improve their knowledge even in fields apart from their own research activity. It allows them to become aware of ongoing scientific progress, creates curiosity and stimulates their critical spirit. Therefore, providing credit to the review activity is important to allow the professional growth of young researchers.

**IH:** Trainees are often the ones writing most of a manuscript or designing and conducting experiments, particularly if it’s for their own dissertation, and sometimes they’re the ones corresponding with the reviewers and journals. Principal investigators also get asked to review a lot of papers not necessarily always within their field of expertise, while trainees rarely get personally asked by journals. If a paper comes along that’s related to some experiment or paper the trainee is first author on, it makes sense the investigator would want their trainee to help write the review. Also, since we never get asked personally by journals, we’d be much more likely to say yes to writing a review. It’s demoralizing to spend a long time on a review and not get credit, so of course you should credit trainees.

**JD:** I was never asked to participate in a peer review by my mentors. Being asked to review a manuscript was an important milestone for me as it indicated that I was accepted as a peer in the scientific community. However, it took some time for me to learn the ins and outs of reviewing, a process that would have been facilitated if my mentors had shared reviewing responsibilities with me. The peer review process is a fundamental aspect of science. Despite this, specific training in the performance of peer review is rare. There is more to peer review than simply critiquing a paper. Not only must the scientific soundness of the work need to be judged, but also the significance of the work, its context in the literature, and its appropriateness for the journal need to be considered. As a mentor, I include my mentees in all reviews that I perform. The mentee benefits from first-hand exposure to the peer review process and the authors and editors benefit from an extra set of eyes assessing the work. But too often the contributions of “junior” reviewers are not acknowledged. Direct recognition of their involvement will benefit trainees’ careers by increasing their recognition as scientists by the scientific community.

*Interviews were conducted by Associate Editor George Inglis*.

